# Mechanically induced Ca^2+^ oscillations in osteocytes release extracellular vesicles and enhance bone formation

**DOI:** 10.1038/s41413-018-0007-x

**Published:** 2018-03-19

**Authors:** Andrea E. Morrell, Genevieve N. Brown, Samuel T. Robinson, Rachel L. Sattler, Andrew D. Baik, Gehua Zhen, Xu Cao, Lynda F. Bonewald, Weiyang Jin, Lance C. Kam, X. Edward Guo

**Affiliations:** 1 Bone Bioengineering Laboratory, Department of Biomedical Engineering, Columbia University, New York, NY USA; 2 Center for Musculoskeletal Research, Department of Orthopaedic Surgery, Johns Hopkins University, Baltimore, MD USA; 3 Indiana Center for Musculoskeletal Health, Indiana University School of Medicine, Indianapolis, IN USA; 4 Microscale Biocomplexity Laboratory, Department of Biomedical Engineering, Columbia University, New York, NY USA

## Abstract

The vast osteocytic network is believed to orchestrate bone metabolic activity in response to mechanical stimuli through production of sclerostin, RANKL, and osteoprotegerin (OPG). However, the mechanisms of osteocyte mechanotransduction remain poorly understood. We’ve previously shown that osteocyte mechanosensitivity is encoded through unique intracellular calcium (Ca^2+^) dynamics. Here, by simultaneously monitoring Ca^2+^ and actin dynamics in single cells exposed to fluid shear flow, we detected actin network contractions immediately upon onset of flow-induced Ca^2+^ transients, which were facilitated by smooth muscle myosin and further confirmed in native osteocytes ex vivo. Actomyosin contractions have been linked to the secretion of extracellular vesicles (EVs), and our studies demonstrate that mechanical stimulation upregulates EV production in osteocytes through immunostaining for the secretory vesicle marker Lysosomal-associated membrane protein 1 (LAMP1) and quantifying EV release in conditioned medium, both of which are blunted when Ca^2+^ signaling was inhibited by neomycin. Axial tibia compression was used to induce anabolic bone formation responses in mice, revealing upregulated LAMP1 and expected downregulation of sclerostin in vivo. This load-related increase in LAMP1 expression was inhibited in neomycin-injected mice compared to vehicle. Micro-computed tomography revealed significant load-related increases in both trabecular bone volume fraction and cortical thickness after two weeks of loading, which were blunted by neomycin treatment. In summary, we found mechanical stimulation of osteocytes activates Ca^2+^-dependent contractions and enhances the production and release of EVs containing bone regulatory proteins. Further, blocking Ca^2+^ signaling significantly attenuates adaptation to mechanical loading in vivo, suggesting a critical role for Ca^2+^-mediated signaling in bone adaptation.

## How bone grows in response to loading

People gain bone in response to exercise and lose it during prolonged bedrest; now we’re closer to understanding how this happens.

Bone cells called osteocytes act as mechanical sensors, responding to changes in force by regulating the activity of bone-forming osteoblasts and bone- resorbing osteoclasts. X. Edward Guo at Columbia University in New York and colleagues had previously shown that osteocytes exhibit oscillations in intracellular calcium in response to mechanical stimulation, but the downstream effects of this had been unclear. Using multiple approaches, they have now shown that the cytoskeleton contracts in response to these oscillations, in turn triggering the production and release of extracellular vesicles containing bone-regulatory proteins.

When calcium signaling was blocked, vesicle production and release was blunted, and mice failed to show the normal increase in bone formation in response to mechanical loading.

## Introduction

The integrity of bone tissue is highly dependent on mechanical stimulation, as evidenced by bone gain in athletes^1,2^ and dramatic bone loss in conditions of extended bedrest^3^ or microgravity^4^. Osteocytes are long-lived, dendritic cells embedded as an interconnected network within the mineralized bone matrix in the lacunar-canalicular system (LCS)^5^. As techniques for isolating and evaluating these cells have advanced, the essential role of osteocytes in bone metabolism has become widely recognized. Osteocytes are the mechanosensors of bone tissue, as well as the primary source of sclerostin^6^, a negative regulator of the bone-forming osteoblasts, and receptor activator of nuclear factor κB ligand (RANKL)^7^, an activating factor of bone-resorbing osteoclasts, both of which are targets for agents evaluated as treatments for osteoporosis^8,9^. Despite surmounting evidence regarding the importance of osteocytes, the mechanisms by which mechanical stimulation influences osteocyte protein production and protein regulation impacts effector cell adaptive responses remain poorly understood.

We have previously shown osteocyte mechanosensitivity to be encoded through unique intracellular calcium (Ca^2+^) dynamics. When exposed to fluid shear in vitro and dynamic deformational loading of whole bones ex vivo, osteocytes exhibit robust, unattenuated oscillations in intracellular Ca^2+^, dependent on extracellular Ca^2+^ and the second messenger, ATP^10,11^. Furthermore, we have shown that release of Ca^2+^ from intracellular stores contributes to these robust responses^12^. Ca^2+^ oscillations in osteocytes are distinct from those of osteoblasts, more abundant than autonomous signals, and become more pronounced under increasing mechanical loading magnitudes^11,13^. Though Ca^2+^ signals can regulate diverse cellular functions in multiple cell types, immediate downstream consequences of mechanosensitive Ca^2+^ oscillations in osteocytes have yet to be identified.

Mechanical stimuli have been shown to influence the osteocyte cytoskeleton. The uniquely organized actin cytoskeleton of osteocytes—from the dense cortical cytoskeleton to the filaments extending along the entire length of dendrite—can respond and restructure in response to mechanical loading^14,15^. In our previous work investigating dynamics of osteocyte cytoskeletal components under flow, we showed that fluid shear introduces strains in the cortical actin network, demonstrating sensitivity of the actin cytoskeleton to mechanical stimuli^16^. Interestingly, the expression of muscle contraction-related proteins is upregulated as bone cells mature into osteocytes^17^. However, though a hallmark of all muscle cells is Ca^2+^-dependent actomyosin contractility, the relationship between Ca^2+^ signals and cytoskeletal dynamics in osteocytes has yet to be explored.

Vesicle release has recently been highlighted as an important means of intercellular communication, whereby cells package proteins and genetic materials in extracellular vesicles (EVs) to shuttle their contents amongst one another^18^. EV release is stimulated by actomyosin contractility in endothelial cells^19^ and facilitated by Ca^2+^/actin dynamics in mast cells^20^. Actin networks have been implicated in mechanically induced protein responses in osteocytes^21^, and vesicle-like structures have also been detected in osteocyte networks in situ^22^. To our knowledge, no studies have explored the regulation of EV production by mechanical loading as a potential mechanism for these observations.

We used a multiscale approach to probe the immediate downstream consequences of mechanically induced osteocyte Ca^2+^ signaling and test the plausibility of osteocyte EV release as a mechanoresponsive means by which osteocytes could coordinate tissue-level adaptation to mechanical loading. We hypothesized that mechanically induced Ca^2+^ signals in osteocytes induce changes in cortical actin dynamics and influence adaptive bone formation, possibly through the release of bone regulatory proteins in EVs.

## Results

**Fig. 1:**
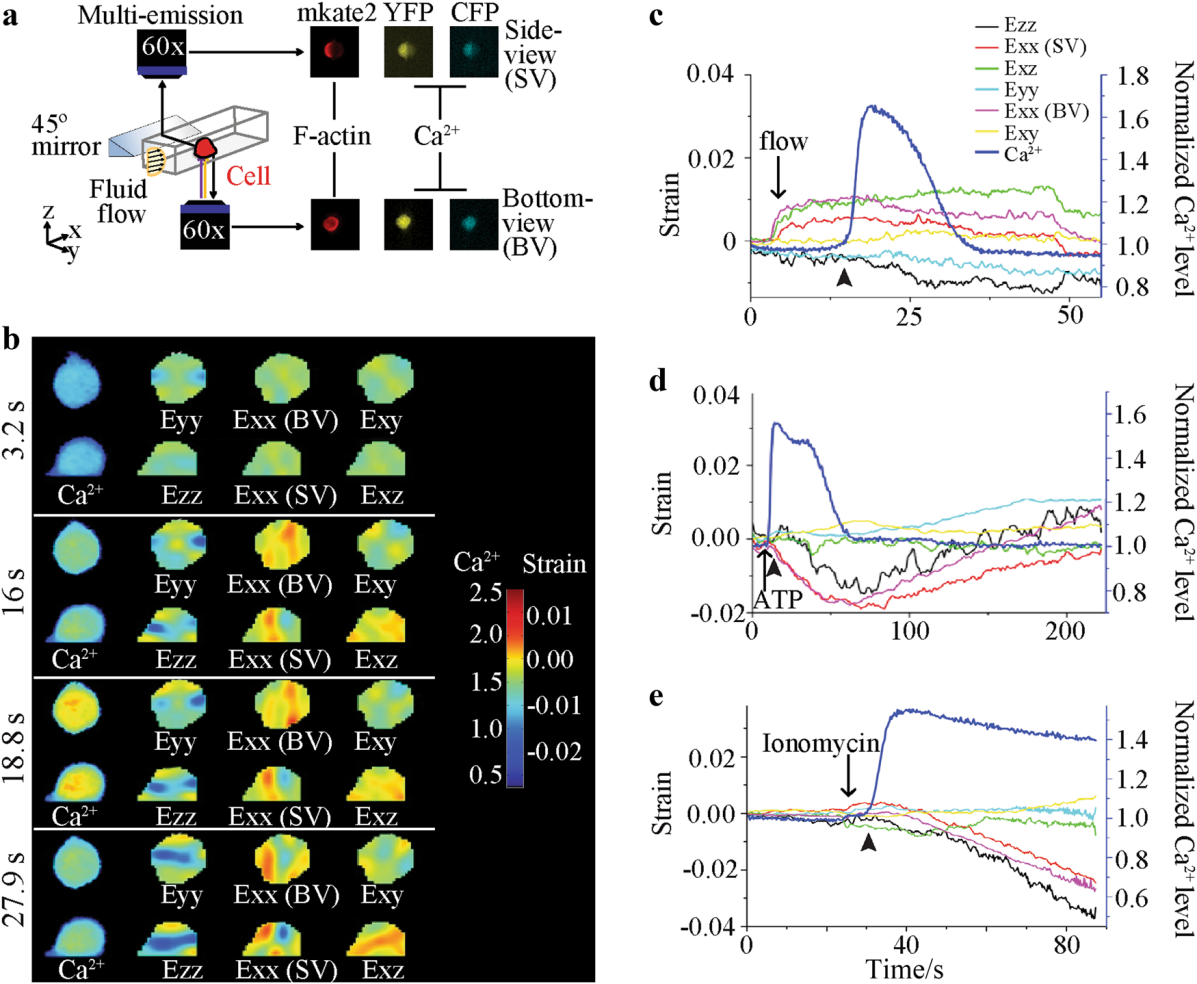
Osteocytes exhibit phasic, Ca^2+^-dependent actin contractions. **a** Quasi-3D multi-emission imaging of single MLO-Y4 cells under flow. **b** Bottom-view and side-view contours of intracellular Ca^2+^ and actin network strains in a single MLO-Y4 osteocyte double-transfected with a Ca^2+^ FRET biosensor and a Lifeact-mkate2 actin probe. **c** Representative traces for actin strains and intracellular Ca^2+^ levels in response to fluid flow, with Ezz (black trace) decreasing immediately after Ca^2+^ initiation (dark blue trace), regardless of any tensile positive strains which developed at the onset of flow. **d** A Ca^2+^ transient induced by ATP results in a phasic contractile response in all normal strains. **e** Ionomycin induces a step-increase in intracellular Ca^2+^ and overall decreases in normal strains. Arrowheads indicate initiation of contractile events.

First, we examined contractility of the actin cytoskeleton as an immediate effect of Ca^2+^ signaling. Ca^2+^ dynamics and actin network strains were simultaneously measured using the multichannel quasi-3D microscopy technique (Fig. 1a). We detected contractions in the actin network of MLO-Y4 cells following the initiation of flow-induced Ca^2+^ transients, as indicated by a decrease in the average intracellular actin normal strain in the height of the cell (Ezz) (Fig. 1b, c). These contractile strains were observed in 70% of the osteocytes that were recorded, regardless of whether flow-induced tensile actin strains preceded Ca^2+^ activation^16^. To further confirm the dependence of actin network contraction on Ca^2+^ dynamics, ATP, a known osteocyte Ca^2+^ signaling mediator^13^, and ionomycin were used to chemically stimulate a Ca^2+^ response in the absence of mechanical load. Within several seconds, compression in all measured normal strains was visible, indicative of a whole-cell contraction (Fig. 1d, e). Ezz height strain tended to have the largest compression, while shear strains were not affected. ATP stimulated cells displayed a recovery to baseline (~180 s), demonstrating a reversible, phasic contraction.

**Fig. 2:**
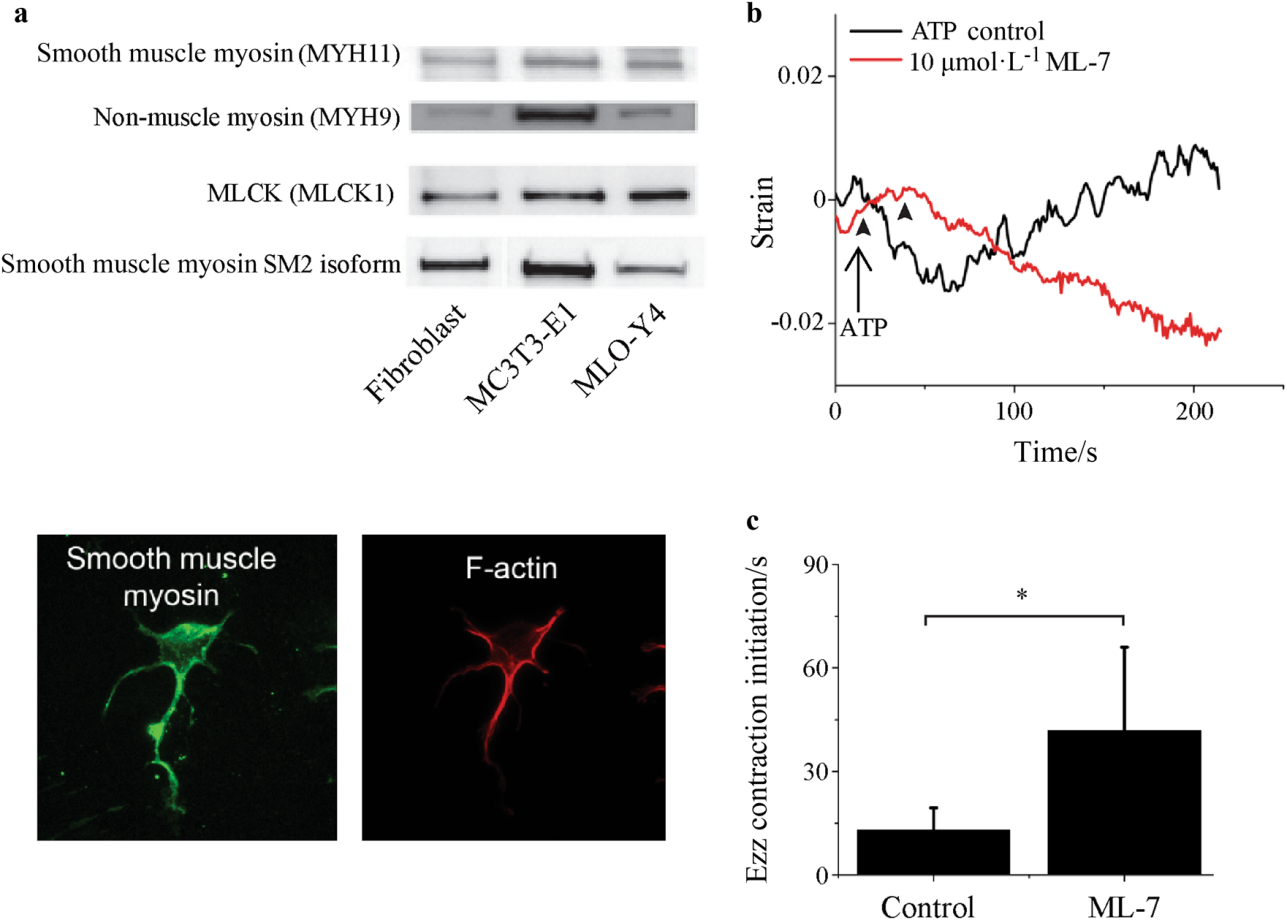
Ca^2+^-dependent contractions in osteocytes are mediated by smooth muscle myosin. **a** Western blots for smooth muscle myosin proteins in NIH3T3 fibroblast cells, osteoblast cell line MC3T3-E1, and osteocyte cell line MLO-Y4. Immunofluorescence in MLO-Y4 cells shows co-localization of smooth muscle myosin with the F-actin network. **b** Representative Ezz trace for MLCK inhibition by ML-7, with a significantly delayed contraction compared to ATP control (see Fig. 1d). **c** The time before a decrease in Ezz strain after ATP induction was increased from (13.3 ± 6.4) s in control cells (*n* = 6) to (41.8 ± 24.2) s in ML-7 treated cells (*n* = 8). Arrowheads indicate initiation of contractile events.*P < 0.05. Error bars are standard deviations.

To explore the mechanism underlying these contractions, we probed osteocytes for smooth, skeletal, and cardiac muscle proteins. In MLO-Y4 cells, smooth muscle myosin-II ATPase and its SM2 isoform were detected along with non-muscle myosin and MLCK via Western blot. Immunodetection of smooth muscle myosin and the actin network in primary osteocytes showed co-localization of the two (Fig. 2a). Western blots failed to detect skeletal muscle contraction-related proteins, including skeletal myosin, skeletal α-actin, skeletal myosin ATPase, or skeletal/cardiac troponins, and all Western blot results were confirmed using lysates of FACS-sorted DMP1topaz(+) cells, verifying that primary osteocytes are similar to MLO-Y4 osteocytes in this regard (data not shown). ATP-stimulated cells were treated with the MLCK inhibitor ML-7 and imaged by quasi-3D to determine its influence on Ca^2+^-dependent contractility (Fig. 2b). With inhibition of MLCK by ML-7, (41.8 ± 24.2) s elapsed before an observed decrease in Ezz strain, compared to (13.3 ± 6.4) s under control conditions (Fig. 2c). Therefore, the kinetics of the Ca^2+^-dependent actomyosin contractions appear to be mediated by smooth-muscle myosin.

**Fig. 3:**
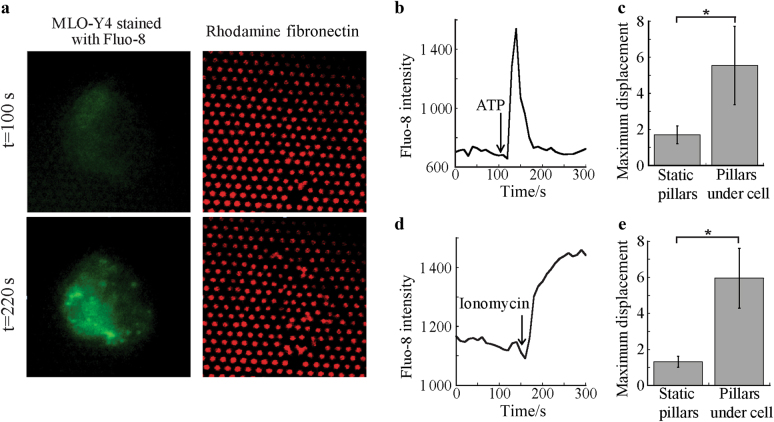
Osteocytes contract micropillar substrates in response to Ca^2+^ induction. **a** Representative MLO-Y4 cell stained with Fluo-8 AM and the underlying micropillars coated with rhodamine fibronectin. Intracellular Ca^2+^ and pillars are shown before and after the addition of ionomycin. **b** Time course of Fluo-8 AM intensity and (**c**) maximum pixel displacement following the addition of ATP of pillars under the cell periphery and static pillars away from the cell (*n* = 3 cells). **d** Time course of Fluo-8 AM intensity and (**e**) maximum pixel displacement following the addition of ionomycin of pillars under the cell periphery and static pillars away from the cell (*n* = 3 cells). *P < 0.05. Error bars are standard deviations.

To verify that decreases in osteocyte actin strains detected in quasi-3D correspond to whole-cell contractions, we utilized a fabricated micropillar assay. Osteocytes were found to exhibit cortical actin arrangement on micropillars, recapitulating the morphology preserved in quasi-3D. Pillar positions were tracked over time after addition of Ca^2+^ agonists to determine displacements following Ca^2+^ induction, indicating cytoskeletal activity (Fig. 3a). Addition of media alone had no effect on pillar displacements. Following addition of ATP (Fig. 3b, c) or ionomycin (Fig. 3d, e), Ca^2+^ was elevated, and pillars near the periphery of the cell deflected further inward, indicating a whole-cell contraction. Particle tracking demonstrated that the maximum displacement of pillars below an osteocyte was significantly higher than static pillars away from the cell in both agonist conditions.

**Fig. 4:**
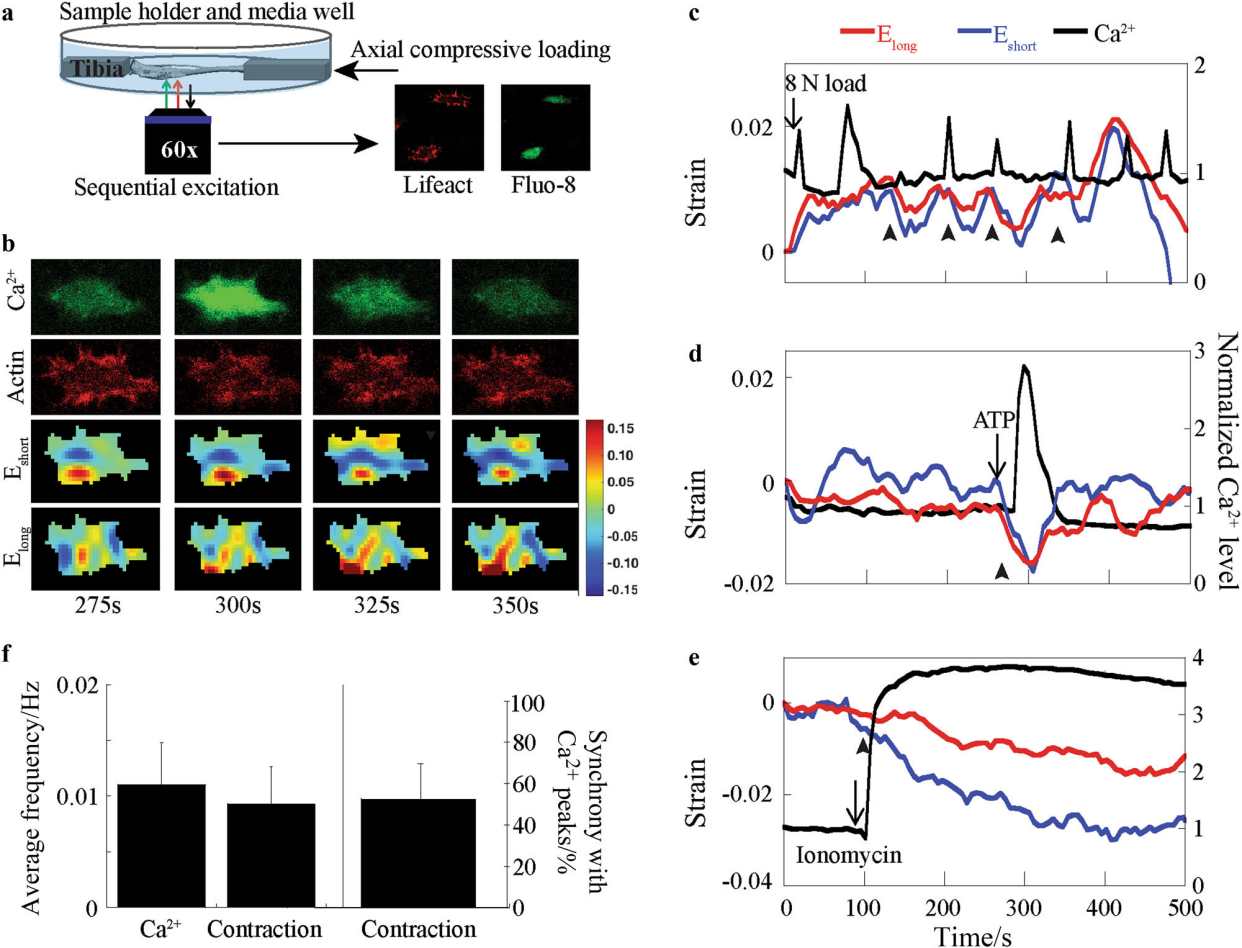
Osteocytes in their native bone matrix environment exhibit Ca^2+^-dependent actin network contractions. **a** Simultaneous loading and imaging of ex vivo osteocytes tagged with Lifeact mRFP and dyed with Fluo-8 AM. **b** Time-course of intracellular Ca^2+^ (Fluo-8 AM intensity), the osteocyte actin network (RFP-tagged F-actin), and whole-cell normal strain contours. **c** Representative traces of actin cytoskeleton strains and Ca^2+^ dynamics in an ex vivo osteocyte in response to cyclic mechanical loading of the tibia at an 8 N load magnitude. Decreasing normal strains develop in both axes of the cell following a Ca^2+^ peak. **d** A Ca^2+^ transient induced by ATP results in a phasic contractile response in normal strains in both axes of the osteocyte. **e** Ionomycin induces a step-increase in intracellular Ca^2+^ and overall decreases in normal strains in both axes of the cell. **f** Quantification of osteocyte Ca^2+^ oscillations and contractile dynamics reveals an average Ca^2+^ spike frequency of (0.012 ± 0.004) Hz and average contraction frequency of (0.009 3 ± 0.003) Hz, which were not found to be significantly different (*n* = 9 cells). Osteocyte contractions exhibit a percent synchrony with Ca^2+^ peaks of (52.4 ± 16.8) %. Arrowheads indicate initiation of contractile events. *P < 0.05. Error bars are standard deviations.

The activation of actin contractions by load-induced Ca^2+^ signaling was further confirmed in osteocytes residing in their physiologic bone matrix in viable murine Lifeact tibiae dyed with Fluo-8 AM, an intracellular calcium indicator dye (Fig. 4a). Decreases in actin network strains occurred along the normal axes of the cells following Ca^2+^ oscillations induced by cyclic compression of the whole bone (Fig. 4b, c). These contractile dynamics exhibited phasic behavior throughout the imaging period. Ca^2+^-dependency of the actin dynamics was again confirmed using ATP and ionomycin (Fig. 4d, e) to elevate intracellular Ca^2+^ levels. The frequencies of the load-induced Ca^2+^ transients and contractions were not found to be significantly different, suggesting their coordination in response to mechanical load. Connecting individual Ca^2+^ peaks with subsequent decreases in strain revealed the majority of Ca^2+^ peaks (>50%) were synchronous with a cytoskeletal contraction (Fig. 4f).

**Fig. 5:**
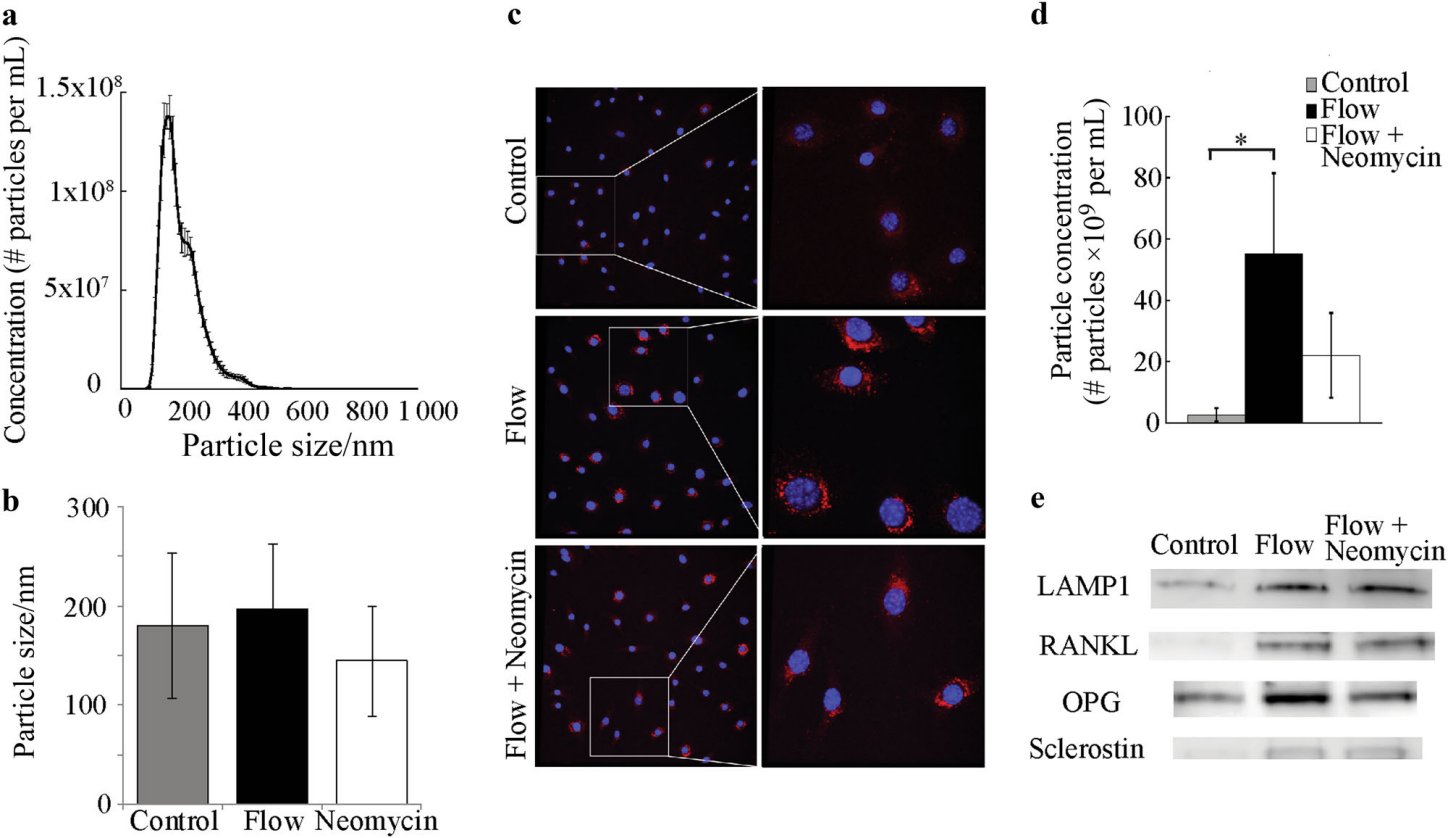
Mechanical stimulation of osteocytes causes a Ca^2+^-dependent release of extracellular vesicles containing bone regulatory proteins. **a** Representative distribution of particle size for a sample of vesicles isolated from cells exposed to flow. **b** Average particle size for each group. **c** Immunostaining for the secretory vesicle marker LAMP1 reveals punctate staining (red) consistent with the presence of vesicle-like structures. Fluid flow increased the expression of LAMP1, while neomycin diminished this response. **d** Nanoparticle concentration analysis shows that fluid flow increased the number of particles released into the medium, and neomycin blunted this response. **e** Western blots on extracellular vesicle protein lysates were probed for the presence of key bone regulatory proteins. Note that lysates were not adjusted for varying vesicle quantities between groups and reflect extracts of total vesicle pellets prepared by ultracentrifugation. *n* = 4 samples/group *P < 0.05. Error bars are standard deviations.

We next sought to investigate a potential connection between Ca^2+^-dependent actomyosin contractions and a downstream function in osteocytes. To determine the role of Ca^2+^-signaling in osteocyte release of EVs, MLO-Y4 cells were exposed to fluid shear both with and without pre-incubation in exosome-depleted medium containing the phospholipase C inhibitor neomycin. EVs isolated from conditioned medium were, on average, a diameter of 175 nm, which did not significantly differ between groups (control, flow, flow + neomycin) (Fig. 5a, b). Immunodetection on cells fixed immediately after stimulation showed increased expression of LAMP1, with more abundant punctate stains visible in cells and present further throughout the cell body. Inhibition of Ca^2+^ oscillations using neomycin visibly diminished this response (Fig. 5c). Mechanical stimulation of MLO-Y4 cells by fluid flow induced substantial release of EVs into the culture medium, and this response was also blunted in the presence of neomycin to levels at which there were no detectable differences between control and flow with neomycin treatment (Fig. 5d). Further, Western blots on osteocyte EV protein lysates controlled by total cell number detected LAMP1 and the bone regulatory proteins RANKL, OPG, and sclerostin (Fig. 5e). Quantitation of these blots was not performed due to the inability to control for exosome number.

**Fig. 6:**
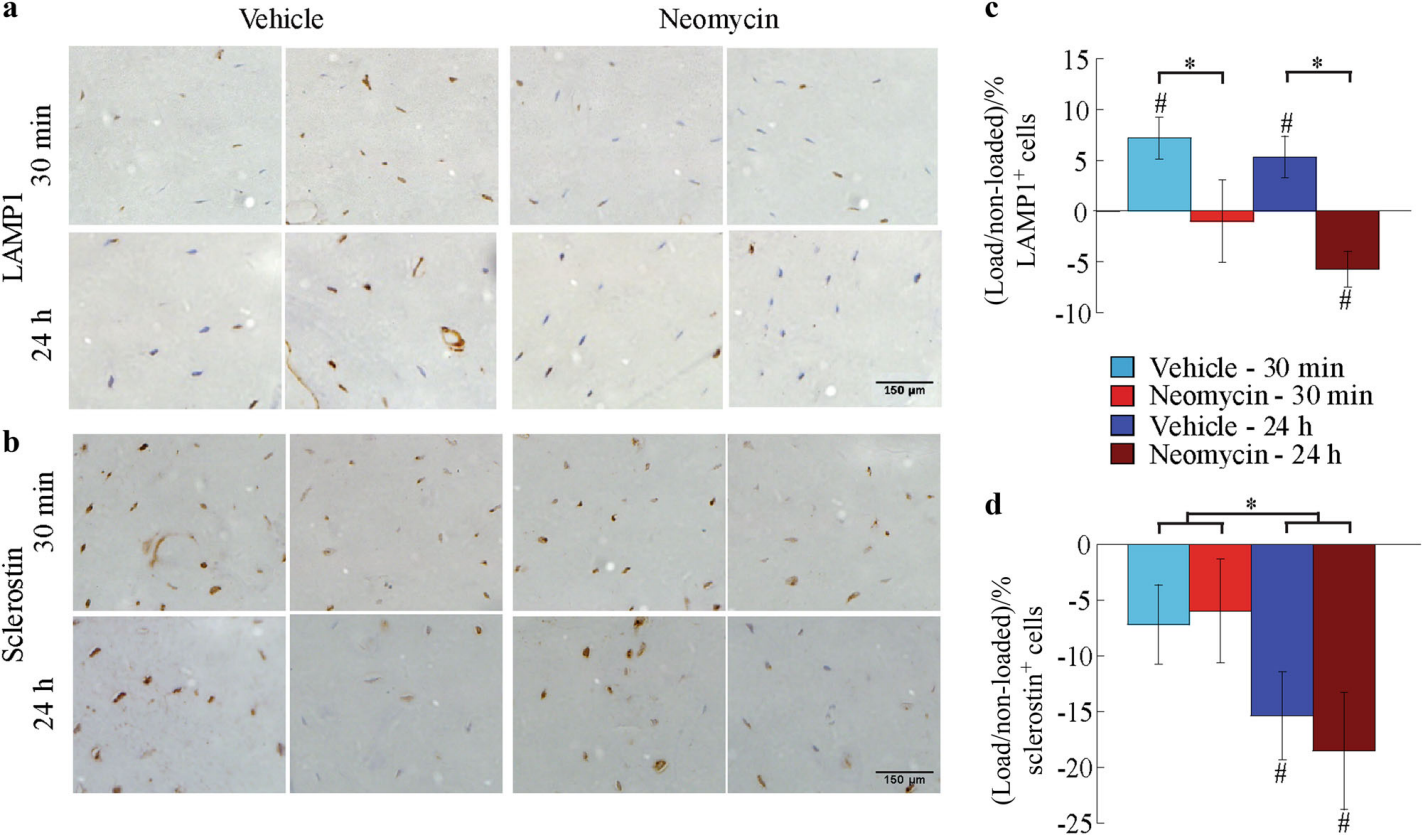
Ca^2+^ oscillations in osteocytes govern load-induced bone adaptation by modulating LAMP1 expression. **a** Representative immunohistochemistry images of LAMP1 and (**b**) sclerostin staining. Brown cell bodies, positive. **c** Average difference in percent of osteocytes with positive staining for LAMP1 or (**d**) sclerostin in vehicle- and neomycin-injected groups sacrificed 30 min or 24 h after the final loading bout. Significant interactions between drug and load in LAMP1 and time and load in sclerostin expression were detected. Within group (#). Between group (*). *n* = 7 mice /group. *P* < 0.05. Error bars are standard errors of the mean.

The Ca^2+^-dependency of actomyosin contractions and EV release in these in vitro and ex vivo studies prompted us to investigate the effect on EV release and bone formation in vivo^23^. Axial tibia compression at anabolic loading levels was performed on C57BL6/J mice. Neomycin injections prior to each loading session were used to antagonize mechanically induced Ca^2+^ responses, which was verified by imaging of Ca^2+^ signaling in ex vivo limbs of neomycin injected mice under strain-matched loading conditions (vs. in vivo strains)^11^, and compared to vehicle controls (Supplemental Fig. 2). To understand the role of EV production in the adaptive response, mice were sacrificed 30 min and 24 h following 2 days of anabolic loading, and immunohistochemistry techniques were used to probe for LAMP1 (Fig. 6a). In addition, sclerostin expression was analyzed to confirm early anabolic loading responses (Fig. 6b). Consistent with our in vitro findings, LAMP1 expression was upregulated in response to mechanical loading in vehicle-treated animals at 30 min and remained significantly elevated in loaded limbs at 24 h. Further, neomycin inhibition eliminated the load-related increase in LAMP1 expression at 30 min and significantly decreased load-related LAMP1 levels at 24 h (Fig. 6c). We detected the expected downregulation of sclerostin expression in loaded limbs at 24 h in vehicle-injected mice, while the load-related downregulation of sclerostin was not altered in neomycin-injected mice (Fig. 6d).

**Fig. 7:**
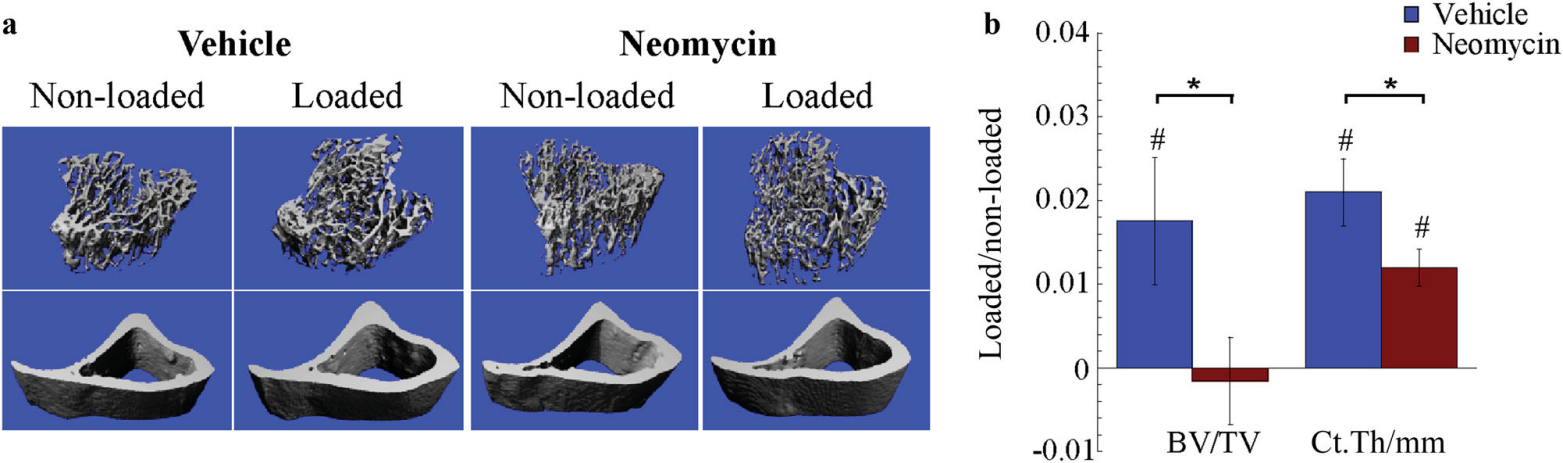
Ca^2+^ oscillations in osteocytes govern load-induced bone adaptation. **a** Representative µCT scans of trabecular and cortical bone in non-loaded and loaded tibiae from animals injected with neomycin or vehicle control. **b** Average difference in bone volume fraction (BV/TV) and cortical thickness (Ct.Th) between loaded and non-loaded control limbs in vehicle- and neomycin-injected mice. Significant decreases in loading-related changes were detected in neomycin-injected group. Within group (#). Between group (*). *n* = 5 mice/group. *P* < 0.05. Error bars are standard errors of the mean.

Finally, we tested whether inhibition of Ca^2+^ oscillations affects load-induced bone adaption. In vivo axial tibia compression was performed on C57BL6/J mice five days per week for two consecutive weeks. Neomycin or vehicle injections were performed one hour before each loading session. Following dissection at the end of the loading period, two scans of each limb were performed to evaluate trabecular and cortical parameters. In line with previous studies, micro-computed tomography of vehicle-injected, loaded and non-loaded limbs revealed significant load-related differences in both trabecular bone volume fraction (BV/TV) and cortical thickness after two weeks (Fig. 7a). Neomycin injections abolished the load-related differences in BV/TV and blunted the cortical thickness response to nearly half that of the vehicle control (Fig. 7b).

## Discussion

The intracellular and intercellular mechanisms by which osteocytes translate changes in the mechanical environment of the bone matrix and orchestrate bone adaptation accordingly via osteoblasts and osteoclasts remain largely unclear. Previous work from our group has suggested that osteocyte mechanosensitivity is encoded through unique intracellular Ca^2+^ dynamics, demonstrated in systems of multiple scales^10–13^. Based on this evidence, we sought to further understand downstream consequences of mechanosensitive Ca^2+^ signaling and its link to mechanotransduction.

Using multiple approaches, both in vitro and ex vivo, we discovered novel, whole cell, actomyosin contractions immediately following Ca^2+^ oscillations in osteocytes. Interestingly, as osteoblasts differentiate into osteocytes, changes in cytoskeleton arrangement and distribution of structural elements occur, including formation of a dense cortical actin shell, as well as actin-enriched structures that extend the entire length of the dendrites^14,24,25^. Furthermore, gene expression related to muscle function, development, and differentiation of skeletal, cardiac, and smooth muscle have been detected in osteocytes^17^. While smooth muscle contractile dynamics have been extensively characterized in other cell types, we are for the first time presenting this Ca^2+^-dependent contractile behavior in osteocytes in a mechanosensitive context.

We further found that mechanically induced Ca^2+^ oscillations also influence the release of EVs by osteocytes. Vesicle-like structures have been detected in osteocyte networks in situ^22^, and osteocyte-like cells have been shown to produce EVs enriched in bone-regulatory miRNAs that can be endocytosed by osteoblasts^26,27^. Though not directly evaluated in this study, our results suggest that actin networks, which have been previously implicated in mechanically induced protein responses in osteocytes^21^, may also play a role in the regulation of EV production by mechanical loading. The technique used to prepare these vesicle fractions from osteocytes as well as the characterization of particle size suggest that these membrane vesicles are likely exosomes or microvesicles^28^. A recent in situ morphological study showed strong co-localization of RANKL to vesicle-like structures positive for LAMP1 in osteocytes^29^ and OPG-positive structures in canaliculi with diameter measurements reported as ~200 nm, in line with previously published values^30,31^. Based on these and our own in vitro findings detecting sclerostin, RANKL, and OPG in mechanically induced EVs, we posit that Ca^2+^-mediated EV release is a plausible means by which osteocytes coordinate tissue-level bone adaptation in response to mechanosensation.

Finally, employing an established model for murine uniaxial tibia loading, we sought to strengthen the link between Ca^2+^ signaling and a functional role in bone adaptation by examining changes in osteocyte protein expression and bone formation response both with and without inhibition of Ca^2+^ signaling. Our immunostaining data for LAMP1, a marker of intracellular secretory vesicles, both in vitro and in vivo suggests that EV production is enhanced with loading, and that osteocyte mechanosensation through Ca^2+^ oscillations plays a role in modulating levels of this EV production. Neomycin abolishment of typical load-induced anabolic bone formation responses in C57BL/6J mice subjected to loading demonstrates that Ca^2+^ signaling is not only essential for mechanosensation, but also key in the long term adaptive response of bone to mechanical loading. Downregulation of sclerostin in response to mechanical loading, on the other hand, appears to be unaltered by neomycin and therefore independent of Ca^2+^-mediated mechanosensation. Sclerostin suppresses bone formation by antagonizing osteoblasts through the canonical-WNT pathway, and multiple studies suggest that sclerostin downregulation is necessary for bone formation to occur^32,33^. Interestingly, we note differences between in vitro release of sclerostin-containing EVs and downregulation of intracellular sclerostin protein expression in osteocytes in vivo, suggesting sclerostin responses to mechanical load may be more nuanced than previously described and warranting further study of this bone regulatory protein and a potential focus on its intercellular transport. Overall, our results demonstrate that inhibition of Ca^2+^ mechanosensation attenuates bone formation despite a downregulation of sclerostin, suggesting that other biochemical signals and mechanisms may be responsible for the full adaptive response.

While our multi-scale approach presents new findings of mechanically induced Ca^2+^oscillations in osteocytes resulting in actomyosin contractions, EV release, and bone adaptive responses, we recognize some limitations and future work necessary to enhance our studies. Ongoing research in our laboratory seeks to experimentally link these observations. For instance, inhibiting osteocyte actomyosin contractions independent of Ca^2+^ signaling would further strengthen the proposed link between the contractions we observe, their influence on osteocyte EV release, and the bone adaptive response. We, along with Solberg and colleagues, acknowledge that the presence of LAMP1 staining in osteocytes does not directly classify these structures as secretory. It would be of future interest to confirm that these structures are indeed EVs via additional markers, as well as identify these structures in the LCS to strengthen their functional role in bone adaptation and cell-cell communication. Further studies are also necessary to determine if and how EVs produced by osteocytes travel along the extended dendritic processes and through the LCS to the bone surface. Of note, it is still unknown how osteocytes send biochemical signals from within the bone matrix to cells on the bone surface, which carry out the adaptive processes we observe here.

This work presents evidence of a novel pathway where osteocytes under mechanical loading can regulate bone metabolism by directly controlling osteocyte-specific protein release through EVs. Avenues of further study of this mechanotransduction mechanism in bone are accessible, as similar mechanisms of Ca^2+^-dependent cell contractions and cytoskeletal influence on EV release have been well studied in other cell types. This provides a new direction to better understand the cellular and molecular mechanisms of bone response to mechanical stimuli, as well as the roles of key bone regulatory proteins, such as RANKL and sclerostin, which may be the target of anti-osteoporosis drugs.

## Materials and methods

### Cell culture

Osteocyte-like MLO-Y4 cells were maintained on collagen-coated tissue culture dishes in minimum essential alpha medium (α-MEM) supplemented with 5% fetal bovine serum and 5% calf serum and kept at 37 °C and 5% CO_2_ in a humidified incubator. Cells were sub-cultured prior to reaching 80% confluency to maintain an osteocyte-like phenotype. To visualize Ca^2+^ and actin simultaneously, MLO-Y4 cells were double transfected using Fugene 6 (Promega, Madison, WI) using the following two plasmids: an improved Cameleon calcium FRET biosensor using ECFP and YPet as donor and acceptor fluorophores^34,35^ and the Lifeact live-cell F-actin probe^36^ cloned into a mkate2 fusion plasmid (Evrogen, Moscow, Russia).

### Quasi-3D microscopy

The quasi-3D microscopy system was described in a previous study from our laboratory^37^. In brief, bottom- and side-view images of a single osteocyte are obtained simultaneously using two EMCCD cameras (Andor, Concord, MA), one coupled to an inverted microscope and the other to an upright microscope with a 45° mirror in the lightpath. A quadview beamsplitter (Photometrics, Tucson, AZ) and custom quad-band polychroic (Chroma, Bellows Falls, VT) allow simultaneous collection of up to 4 fluorescent emissions. Transfected MLO-Y4 cells were plated on fibronectin-coated glass microslides for 45 min to ensure a fully attached, rounded cell shape with proper cortical F-actin networks in the cell body. Microslides were then inserted into a square glass tube (VitroCom, Mountain Lakes, NJ) for imaging and fluid shear stimulation. For mechanically induced Ca^2+^ responses, steady, unidirectional fluid flow (*n* = 10) was applied at 2 Pa using a syringe pump (Kent Scientific, Torrington, CT). For biochemical-induced Ca^2+^ responses, ATP (*n* = 6, 50 µmol·L^−1^) or ionomycin (*n* = 5, 5 µmol·L^−1^) (Sigma-Aldrich, St. Louis, MO) were introduced into the imaging chamber at a low flow rate to ensure a wall shear stress less than 0.075 Pa. Cells were pre-incubated with ML-7 (*n* = 8, 10 µmol·L^−1^, Sigma-Aldrich) for 20 min before ATP injection to inhibit myosin light chain kinase (MLCK). All image analysis was performed in MATLAB 7.8 (The Mathworks, Natick, MA). Intracellular displacement fields of the actin were obtained for both views using a zero order digital image correlation technique. The *x*-direction was defined as the flow direction, and the *y*-direction and *z*-direction as the lateral and height directions, respectively. Finite Lagrangian strain fields were calculated from the displacement fields using a bilinear least squares filter followed by a thin-plate spline differentiation to give three bottom-view strains: Eyy, bottom-view Exx, and shear Exy; and three side-view strains: Ezz, side-view Exx, and shear Exz. ECFP, and YPet FRET images were aligned using a normalized cross-correlation algorithm and YPet/ECFP emission ratios were calculated for each timepoint.

### Western blots

Cells were lysed in RIPA buffer supplemented with a protease/phosphatase cocktail (ThermoScientific, Waltham, MA) and quantified using a Bio-Rad protein quantification assay (Bio-Rad, Hercules, CA). Ten microgram of protein was loaded into 7.5% or 12% Tris-HCl SDS-PAGE electrophoresis gels and transferred overnight onto PVDF membranes. Primary antibodies raised in rabbit for smooth muscle myosin heavy chain (MYH11), non-muscle myosin heavy chains (MYH9), myosin light chain kinase (MLCK1), and smooth muscle myosin heavy chain SM2 (MYH11) were obtained from Abcam (Abcam, Cambridge, MA). Secondary anti-rabbit antibodies conjugated to horseradish peroxidase were obtained from Abcam. Gels were imaged using ECL^+^ (GE Healthcare Biosciences, Pittsburgh, PA) and a CCD blot imager (Nikon, Japan). NIH3T3 protein lysates were used as positive controls for smooth muscle proteins (Abcam).

### Micropillar substrates

MLO-Y4 cells stained with the Ca^2+^ indicator Fluo-8 AM (AAT Bioquest, Sunnyvale, CA) dissolved in 20% Pluronic F-127 in DMSO (Invitrogen, Carlsbad, CA) were seeded onto rhodamine-fibronectin (Cytoskeleton, Inc, Denver, CO) coated micropillars fabricated from PDMS with 1 µm diameter and 7 µm height^38,39^. Cells were seeded on the pillars for 45 min to maintain a cortical actin cytoskeleton. Particle tracking was performed using a threshold-based algorithm to determine the maximum displacement of pillars underneath an osteocyte following a Ca^2+^ response induced by ATP (50 µmol·L^−1^, *n* = 3) or ionomycin (5 µmol·L^−1^, *n* = 3) infused gently into the dish with a syringe. Static pillars away from the cell were tracked as a control, and medium without inhibitors was added as additional control sample.

### Animals

C57BL/6 mice were purchased from The Jackson Laboratory (Bar Harbor, ME). Lifeact mRFPruby mice were a gift from Michael Sixt and Roland Wedlich-Söldner at the Max Planck Institute of Biochemistry^40^ and were bred with C57BL/6 J mice to produce offspring with the filamentous actin (F-actin) network tagged with mRFP in all cell types, including osteocytes. Lifeact genotyping was performed by screening tail-snipped tissue samples for RFP. All animals were housed 3–5/cage and given access to food and water *ad libitum* (PicoLab 5053 and 5058, LabDiet, St. Louis, MO). All animal procedures were approved by the Institutional Animal Care and Use Committee at Columbia University in accordance with Institute for Comparative Medicine and national guidelines.

### Ex vivo fluorescent imaging of osteocytes

Imaging of ex vivo osteocytes in live murine long bone was performed as previously described^11^. Bilateral tibiae were dissected from 3-month-old Lifeact mice immediately after euthanasia and incubated at 37 °C in α-MEM supplemented with 5% fetal bovine serum (FBS), 5% calf serum, and 1% penicillin streptomycin for 2 h. Tibiae were incubated with 20 µmol·L^−1^ Fluo-8 AM for 45 min, washed, and post-incubated for 15 min. Samples were transferred to a custom loading device and submerged in supplemented α-MEM (5% charcoal-stripped FBS) for imaging. Both calcium signaling (488 nm) and actin network dynamics (512 nm) of the ex vivo osteocytes were imaged using an Olympus FluoView FV1000 laser scanning confocal microscope with a ×60 long working distance objective (Olympus, Waltham, MA). Osteocytes were identified ~30 μm below the bone surface and sequentially imaged at 1.1 s/frame for each of the two fluorescent channels. To confirm the Ca^2+^-dependency of the actin network response, either 20 mmol·L^−1^ ATP or 20 µmol·L^−1^ ionomycin was added post-baseline image acquisition to the tibia secured in the custom loading device while Ca^2+^ and actin network dynamics were imaged simultaneously. Asynchronous mechanical loading and imaging of ex vivo osteocytes in murine long bone was performed as previously described^11^. Briefly, compressive cyclic loading was applied axially to the tibia using a linear actuator (M-227.10; Physik Instrumente, Karlsruhe, Germany) and measured by a 5-lb load cell (Model 31; Honeywell, Columbus, OH) to produce triangular load waveforms with an 8 N magnitude. A dwell time of 6 s was applied between loading cycles to allow for image acquisition. Time-lapse image stacks of the actin network were analyzed using MATLAB to directly estimate strain fields and calculate average strains of the cytoskeletal network relative to the long and short axes of the osteocyte cell body over time^41^. Average pixel intensity of Ca^2+^ in each individual cell was normalized by the average intensity of corresponding baseline frames and detrended by subtracting a best-fit 6th order polynomial. A Ca^2+^ spike was defined as a transient increase greater than five times the magnitude of the baseline intensity noise in each cell. A contraction in either the long or short axis of the cell was defined by a minimum prominence of five times the standard deviation of the noise of the baseline strain of each cell. The frequency of Ca^2+^ spikes and contractions in both the long and short axes of the cells were calculated for 9 cells total with >2 Ca^2+^ spikes from 4 different tibiae. A measure of percent synchrony between the contractions in each of the cell axes and Ca^2+^ spikes was calculated as described previously^12^.

### Isolation and characterization of extracellular vesicles

MLO-Y4 cells were plated onto 10 μg·mL^−1^ fibronectin-coated (Corning, Corning, NY) large glass slides (38 × 75 mm) at a density of 30 × 10^4^ cells/slide and cultured for 36 h. Prior to fluid shear exposure, cells were rinsed 3 times for 5 min each in PBS and pre-incubated with minimum α-MEM supplemented with exosome-depleted FBS (System Biosciences, Palo Alto, CA) to remove contaminating bovine exosomes. For the inhibitory group, 15 mmol·L^−1^ of neomycin (Sigma-Aldrich), shown in earlier studies of micropatterned cells to reduce the average number of Ca^2+^ transients to approximately a single response^13^, was added to exosome-free medium. We have confirmed that neomycin has a similar effect on Ca^2+^ signaling in MLO-Y4 cells grown to confluency and exposed to fluid flow (Supplemental Fig. 1)^12^. Slides were assembled into a custom parallel-plate flow chamber, and fluid shear was applied at 35 dynes/cm^2^ for two 10-minute bouts of steady flow separated by a 15 min rest period, which has been shown to induce multiple Ca^2+^ responses in osteocytes in our previous studies^12,13^. Control slides were sealed in dishes with equal volume of exosome-free medium and maintained outside of the incubator during the steady flow experiments. Conditioned medium from each respective group was collected immediately after the second bout of flow and frozen at −20 °C prior to analysis. Exosomes were purified from conditioned medium by differential ultracentrifugation^42^ on a Beckman L8-M ultracentrifuge (Beckman Coulter, Brea, CA) with a 50.2 Ti fixed-angle rotor. Pellets from six independent slides exposed to one of the experimental conditions (control, steady flow, or steady flow with neomycin treatment) were combined for analysis and re-suspended in 1 mL PBS for particle characterization. Particle concentration and size distribution were analyzed by Nanoparticle Tracking Analysis on a Malvern NanoSight (Malvern, United Kingdom) at the Cornell Nanobiotechnology Center. Data from 5 separate measurements of the same sample were averaged to determine the sample concentration and average particle size. The average values for *n* = 4 samples per group were compared. Exosome contents were assessed by Western blot. Exosome protein lysates were prepared by lysing vesicle preparations in RIPA lysis and extraction buffer supplemented with protease and phosphatase inhibitors for 10 min. Equivalent volumes of the exosome suspension prepared for each group (normalized by total cell number) were used to compare among groups. Protein lysates were mixed 1:1 with 2 × Laemmli buffer (Sigma-Aldrich) and boiled for 5 min at 95 °C. Protein samples were separated by gel electrophoresis using pre-cast polyacrylamide gels and a Mini-PROTEAN electrophoresis chamber (Biorad, Hercules, CA). Proteins were transferred to a PVDF membrane (Biorad) by wet transfer. Membranes were blocked with 5% BSA (Sigma-Aldrich), and primary antibody incubations were performed overnight at 4 °C. The following primary antibodies were used to assess vesicle contents: goat polyclonal antibody to RANKL (Santa Cruz Biotechnology, Dallas, TX); goat polyclonal antibody to OPG (Santa Cruz); rabbit polyclonal antibody to lysosomal associated membrane protein 1 LAMP1 (Abcam); goat polyclonal to sclerostin (R&D Systems, Minneapolis, MN). Membranes were rinsed well with TBST and then probed with the appropriate secondary antibodies. Detection was performed using the SuperSignal West Femto chemiluminescence detection kit (ThermoScientific) and a FujiFilm LAS-4000 Luminescent Image Analyzer (FujiFilm, Stamford, CT).

### Immuno-detection

For smooth muscle mechanism probing, Lifeact-transfected MLO-Y4 cells were fixed in formalin, permeabilized in 0.25% Triton X-100, and blocked in 1% BSA. Cells were incubated with an anti-smooth muscle myosin heavy chain 11 primary antibody (Abcam) for 1 h at room temperature, then with an Alexa Fluor 488 secondary antibody (Invitrogen). For imaging of EV release, cells were probed for the expression of the secretory vesicle marker LAMP1 using standard immunocytochemistry techniques. Immediately following flow exposure, MLO-Y4 cells were fixed and permeabilized in ice-cold acetone. Cells were blocked in 5% BSA and incubated overnight with a rabbit polyclonal anti-LAMP1 antibody (Abcam). The VectaFluor Detection system with DyLight 594 Anti-rabbit IgG reagent (Vector Laboratories, Burlingame, CA) was used to probe the antibody for LAMP1. Images were captured on a FV1000 confocal microscope.

### Uniaxial tibia loading and Ca^2+^ inhibition

Male, 12-week-old C57BL/6 J mice were randomly divided into short-term (*n* = 7/group) and long-term (*n* = 5/group) loading groups. In both studies, the right tibia of each mouse was loaded using a non-invasive uniaxial Bose Biodynamic 5500 loading system (Bose Corporation, Framingham, MA). The loading protocol featured a sinusoidal profile ranging between a compressive 2 N preload and a 12 N peak for 100 cycles at 2 Hz frequency. To test the effects of Ca^2+^ transients on short-term protein response to loading and long-term tissue adaptation, half of the mice in each study received a neomycin injection (100 mg·kg^−1^) one hour prior to each loading session, while the other half received the vehicle alone. The short-term loading group was loaded for 2 consecutive days and the long-term loading group mice were loaded 5 days/week for 2 weeks. Mice in the short-term loading group were sacrificed either 30 minutes or 24 h following the final loading session. Mice in the long-term loading group were sacrificed 24 h after the final loading session.

### Immunohistochemistry

Upon sacrifice, cardiac perfusion was performed, after which both tibiae were dissected and fixed in 10% buffered formalin for 48 h. Tibiae were then rinsed and decalcified in 10% EDTA at 4 °C for 2 weeks. Once decalcified, tibiae were processed for routine paraffin embedding. Four-micron transverse sections were cut from the same cortical region analyzed by μCT. Prepared slides were deparaffinized in xylenes, rehydrated through graded ethanol, and heat-mediated antigen retrieval was performed to unmask antigens. Blocking of endogenous peroxidase activity was performed (0.1% sodium azide with 3% H_2_O_2_ in dH_2_O), followed by protein block for 1 h (0.3% Triton X-100 in 4% BSA). Sections were then treated overnight at 4 °C with antibodies against sclerostin (1 μg·mL^−1^, Abcam) or LAMP1 (2.5 μg·mL^−1^, Abcam). Detection was performed using the Vectastain ABC HRP kit and developed with the ImmPACT DAB Peroxidase (HRP) Substrate kit (Vector Laboratories). Hematoxylin was used as a counterstain (Sigma-Aldrich). Slides were then dehydrated, cleared with xylenes and cover slipped using Permount (Fisher Scientific, Pittsburgh, PA). Proximal tibial sections containing portions of growth plate and articular cartilage were processed identically and served as positive controls, as hypertrophic chondrocytes also stain for LAMP1 and sclerostin in these respective regions^29,43,44^. Negative controls were sections processed simultaneously with no primary antibody in diluent for that step. For quantification, the interosseous crest (IC) of transverse sections was imaged live using a ×60 oil-immersion objective and Osteomeasure (Osteometrics, Atlanta, GA) was employed to mark all positive and negative osteocytes in this region. The largest compressive loads, load-related change in sclerostin expression, and increases in cortical thickness have all been previously shown to occur at the IC in the mid-shaft of the tibia^32,33^. Three, blinded observers recorded both total number and positive osteocytes (observed with brown staining in >50% of cell) in each section, and 3 or 4 non-consecutive sections were counted from each tibia for each protein. The average percent of positive osteocytes from the three observations was obtained for each section, and the average of all sections from a single tibia was used for final comparisons. Comparisons were made between the loaded and non-loaded limb for each animal.

### Micro-computed tomography (μCT)

Trabecular and cortical morphological parameters were assessed via micro-computed tomography using a Scanco VivaCT 40 system (Scanco Medical AG, Brüttisellen, Switzerland). Scanning parameters were 55 kVp energy, 109 µA intensity, and 300 ms integration time; reconstructed images had 10.5 µm isotropic voxel size. Prior to all analyses, a Gaussian filter was applied (sigma = 0.8, support = 1) to reduce noise. The trabecular region of interest was defined starting approximately 50 µm below the growth plate and extending 1.05 mm distally. The cortical region of interest was set at 1.05 mm in length, centered midway between the tibial plateau and the distal tibiofibular junction. This reproducible landmark has been shown previously to be the site of highest strains in uniaxial tibia loading^45^. Global thresholds of 30% and 35% of the maximum gray scale value were used to classify trabecular and cortical bone, respectively. Standard Scanco evaluation software was used in the calculation of output parameters.

### Statistical analysis

Student’s *t*-tests were performed to compare differences in means for cell contraction initiation and micropillar displacement. Comparisons between ex vivo cell dynamic frequencies and between percent synchrony measures were performed using one-way ANOVA, with further comparisons made using a Student’s *t*-test on paired data for each individual cell. One-way ANOVA with Dunnett’s post hoc was used to compare differences in means for the nanoparticle concentration (EV release) among the flow and flow + neomycin groups relative to the control group. A three-way mixed ANOVA was run to understand effects of time, drug, and loading on sclerostin and LAMP1 expression. Paired *t*-tests were performed to determine the load-related difference in bone parameters and percentage of positive osteocytes within the vehicle or neomycin group for in vivo studies. Student’s *t*-tests were performed to compare load-related response between vehicle and neomycin injected groups for individual bone parameters. Statistical significance was considered at *P* < 0.05 (IBM SPSS Statistics for Windows v.23, Armonk, NY).

## Electronic supplementary material

Reviewer 1 Comments to the Author

## References

[1] Haapasalo H (2000). Exercise-induced bone gain is due to enlargement in bone size without a changein volumetric bone density: a peripheral quantitative computed tomography studyof the upper arms of male tennis players. Bone.

[2] Taaffe DR, Robinson TL, Snow CM, Marcus R (1997). High-impact exercise promotesbone gain in well-trained female athletes. J. Bone Miner. Res..

[3] Kazakia GJ (2014). The influence of disuse on bone microstructure and mechanics assessed by HR-pQCT. Bone.

[4] Smith SM (2014). Fifty years of human space travel: implications for bone and calcium research. Annu. Rev. Nutr..

[5] Bonewald LF (2011). The amazing osteocyte. J. Bone Miner. Res..

[6] Burgers TA, Williams BO (2013). Regulation of Wnt/β-catenin signaling within and from osteocytes. Bone.

[7] O’Brien CA, Nakashima T, Takayanagi H (2013). Osteocyte control of osteoclastogenesis. Bone.

[8] Spyropoulou A, Karamesinis K, Basdra EK (2015). Mechanotransduction pathways in bone pathobiology. Biochim. Et. Biophys. Acta.

[9] Khosla S, Shane E (2016). A Crisis in the Treatment of Osteoporosis. J. Bone Mineral. Res..

[10] Lu XL, Huo B, Park M, Guo XE (2012). Calcium response in osteocytic networks under steady and oscillatory fluid flow. Bone.

[11] Jing D (2014). In situ intracellular calcium oscillations in osteocytes in intact mouse long bones under dynamic mechanical loading. FASEB J..

[12] Brown GN, Leong PL, Guo XE (2016). T-Type voltage-sensitive calcium channels mediate mechanically-induced intracellular calcium oscillations in osteocytes by regulating endoplasmic reticulum calcium dynamics. Bone.

[13] Lu XL, Huo B, Chiang V, Guo XE (2012). Osteocytic network is more responsive in calcium signaling than osteoblastic network under fluid flow. J. Bone Mineral Res..

[14] Tanaka-Kamioka K, Kamioka H, Ris H, Lim SS (1998). Osteocyte shape is dependent on actin filaments and osteocyte processes are unique actin-rich projections. J. Bone Mineral Res..

[15] Vatsa A (2008). Osteocyte morphology in fibula and calvaria–is there a role for mechanosensing?. Bone.

[16] Baik AD, Qiu J, Hillman EM, Dong C, Edward Guo X (2013). Simultaneous tracking of 3D actin and microtubule strains in individual MLO-Y4 osteocytes under oscillatory flow. Biochem. Biophys. Res. Commun..

[17] Paic F (2009). Identification of differentially expressed genes between osteoblasts and osteocytes. Bone.

[18] Théry C (2011). Exosomes: secreted vesicles and intercellular communications. F1000 Biol. Rep..

[19] Nightingale TD (2011). Actomyosin II contractility expels von Willebrand factor from Weibel–Palade bodies during exocytosis. J. Cell Biol..

[20] Wollman R, Meyer T (2012). Coordinated oscillations in cortical actin and Ca2 + correlate with cycles of vesicle secretion. Nat. Cell Biol..

[21] McGarry JG, Klein-Nulend J, Prendergast PJ (2005). The effect of cytoskeletal disruption on pulsatile fluid flow-induced nitric oxide and prostaglandin E2 release in osteocytes and osteoblasts. Biochem. Biophys. Res. Commun..

[22] Kamel-ElSayed SA, Tiede-Lewis LM, Lu Y, Veno PA, Dallas SL (2015). Novel approaches for two and three dimensional multiplexed imaging of osteocytes. Bone.

[23] Jepsen KJ, Silva MJ, Vashishth D, Guo XE, van der Meulen MC (2015). Establishing biomechanical mechanisms in mouse models: practical guidelines for systematically evaluating phenotypic changes in the diaphyses of long bones. J. Bone Mineral Res..

[24] Kamioka H, Sugawara Y, Honjo T, Yamashiro T, Takano-Yamamoto T (2004). Terminal differentiation of osteoblasts to osteocytes is accompanied by dramatic changes in the distribution of actin-binding proteins. J. Bone Mineral Res..

[25] Murshid SA (2007). Actin and microtubule cytoskeletons of the processes of 3D-cultured MC3T3-E1 cells and osteocytes. J. Bone Mineral Metab..

[26] Williams AL (2017). C5 inhibition prevents renal failure in a mouse model of lethal C3 glomerulopathy. Kidney Int..

[27] Qin Y (2017). Myostatin inhibits osteoblastic differentiation by suppressing osteocyte-derived exosomal microRNA-218: A novel mechanism in muscle-bone communication. J. Biol. Chem..

[28] Gyorgy B (2011). Membrane vesicles, current state-of-the-art: emerging role of extracellular vesicles. Cell. Mol. life Sci..

[29] Solberg LB, Stang E, Brorson SH, Andersson G, Reinholt FP (2015). Tartrate-resistant acid phosphatase (TRAP) co-localizes with receptor activator of NF-KB ligand (RANKL) and osteoprotegerin (OPG) in lysosomal-associated membrane protein 1 (LAMP1)-positive vesicles in rat osteoblasts and osteocytes. Histochem. Cell Biol..

[30] Sharma D (2012). Alterations in the osteocyte lacunar-canalicular microenvironment due to estrogen deficiency. Bone.

[31] Wang L (2005). In situ measurement of solute transport in the bone lacunar-canalicular system. Proc. Natl Acad. Sci. USA.

[32] Robling AG (2008). Mechanical stimulation of bone in vivo reduces osteocyte expression of Sost/sclerostin. J. Biol. Chem..

[33] Moustafa A (2012). Mechanical loading-related changes in osteocyte sclerostin expression in mice are more closely associated with the subsequent osteogenic response than the peak strains engendered. Osteoporos. Int..

[34] Ouyang M, Sun J, Chien S, Wang Y (2008). Determination of hierarchical relationship of Src and Rac at subcellular locations with FRET biosensors. Proc. Natl Acad. Sci. USA.

[35] Miyawaki A (1997). Fluorescent indicators for Ca2 + based on green fluorescent proteins and calmodulin. Nature.

[36] Riedl J (2008). Lifeact: a versatile marker to visualize F-actin. Nat. Methods.

[37] Baik AD (2010). Quasi-3D cytoskeletal dynamics of osteocytes under fluid flow. Biophys. J..

[38] Bashour KT (2014). CD28 and CD3 have complementary roles in T-cell traction forces. Proc. Natl Acad. Sci. USA.

[39] Tan JL (2003). Cells lying on a bed of microneedles: an approach to isolate mechanical force. Proc. Natl Acad. Sci. USA.

[40] Riedl J (2010). Lifeact mice for studying F-actin dynamics. Nat. Methods.

[41] Boyle JJ (2014). Simple and accurate methods for quantifying deformation, disruption, and development in biological tissues. J. R. Soc. Interface.

[42] Thery C., Amigorena S., Raposo G., Clayton A. Isolation and characterization of exosomes from cell culture supernatants and biological fluids. *Curr. Protocols Cell Biol.* 3–22 (2006). 10.1002/0471143030.cb0322s30 18228490

[43] Thompson ML, Jimenez-Andrade JM, Mantyh PW (2016). Sclerostin immunoreactivity Increases in cortical bone osteocytes and decreases in articular cartilage chondrocytes in aging mice. J. Histochem. Cytochem..

[44] van Bezooijen RL (2009). Sclerostin in mineralized matrices and van Buchem disease. J. Dent. Res.

[45] De Souza RL (2005). Non-invasive axial loading of mouse tibiae increases cortical bone formation and modifies trabecular organization: a new model to study cortical and cancellous compartments in a single loaded element. Bone.

