# Supplementary material for: Mechanically induced Ca2+ oscillations in osteocytes release extracellular vesicles and enhance bone formation
Source: Bone Res. 2018 Mar 19;6:6. doi: 10.1038/s41413-018-0007-x (PMC5859015; doi:10.1038/s41413-018-0007-x)
Supplement: Supplementary file 1 — Reviewer 1 Comments to the Author [file 41413_2018_7_MOESM1_ESM.docx]

**Supplemental Material**

Supplemental Figure S1.

Inhibition of Ca^2+^ oscillations by neomycin. (A) Representative traces for MLO-Y4 cells stained with the Fluo-8 Ca^2+^ indicator and exposed to steady flow at t=60s under control conditions or with 15mM neomycin treatment for 15 minutes prior to flow. The drug was also included in the flow medium. (B) Average number of Ca^2+^ transients per cell in responsive cells under control conditions or with 15mM neomycin treatment. (C) Distribution of number of Ca^2+^ peaks within cells under control conditions or with 15mM neomycin treatment. Student’s t-test .*p<0.05. Error bars are standard deviations.

Supplemental Figure S2.

Neomycin injections reduce the number of Ca^2+^ peaks in murine tibia. (A) Representative traces for *ex vivo* osteocytes in tibiae from mice injected with either PBS vehicle or 100mg/kg neomycin (n=5/group). (B) Average number of Ca^2+^ peaks in responsive *ex vivo* osteocytes in tibiae from mice injected with either PBS vehicle or neomycin. Student’s t-test, p=0.0756. Error bars are standard deviations.
